# Microparticles and Nanoparticles from Plants—The Benefits of Bioencapsulation

**DOI:** 10.3390/vaccines9040369

**Published:** 2021-04-11

**Authors:** Jennifer Schwestka, Eva Stoger

**Affiliations:** Institute of Plant Biotechnology and Cell Biology, Department of Applied Genetics and Cell Biology, University of Natural Resources and Life Sciences, 1190 Vienna, Austria; jennifer.schwestka@boku.ac.at

**Keywords:** bio-encapsulation, plant molecular farming, microparticles, protein bodies, virus-like particles, drug delivery vehicle

## Abstract

The efficacy of drugs and vaccines depends on their stability and ability to interact with their targets in vivo. Many drugs benefit from encapsulation, which protects them from harsh conditions and allows targeted delivery and controlled release. Although many encapsulation methods are inexpensive, such as the formulation of tablets for oral delivery, others require complex procedures that add significantly to production costs and require low-temperature transport and storage, making them inaccessible in developing countries. In this review we consider the benefits of encapsulation technologies based on plants. Plant-derived biopolymers such as starch and the maize storage protein zein are already used as protective coatings, but plant cells used as production host provide natural in vivo bioencapsulation that survives passage through the stomach and releases drugs in the intestine, due to the presence of microbes that can digest the cell wall. Proteins can also be encapsulated in subcellular compartments such as protein bodies, which ensure stability and activity while often conferring additional immunomodulatory effects. Finally, we consider the incorporation of drugs and vaccines into plant-derived nanoparticles assembled from the components of viruses. These are extremely versatile, allowing the display of epitopes and targeting peptides as well as carrying cargoes of drugs and imaging molecules.

## 1. Introduction

The ability of drugs to interact with specific targets is facilitated by the use of appropriate carriers, known as drug delivery vehicles (DDVs). The most suitable DDV depends on the selected delivery route. Although many drugs are injected directly into the systemic circulation, others are administered topically or orally, the latter often preferred because it is simple and noninvasive, and dosing is easy to control [1]. However, oral delivery places additional demands on the drug, such as oral bioavailability, ability to withstand stomach acid, and resistance to digestive enzymes. If these hurdles cannot be overcome, a different administration route is necessary [2]. Syringe-assisted administration requires trained staff, high standards of hygiene, and typically a cold chain for drug transport and storage, all of which are expensive and place a disproportionate burden on developing countries with incomplete healthcare infrastructure. This has prompted research into new DDVs that facilitate mucosal delivery, typically via the oral and nasal routes.

All drugs encounter some barriers before they reach their site of action, and the role of the DDV is to overcome such barriers while protecting the drug and, if necessary, avoiding premature release that may cause off-target effects. The encapsulation of drugs not only provides protection but can also achieve additional useful functions, such as the enhancement of solubility or controlled release. In recent years for instance, the nanoencapsulation of poorly water-soluble bioactive substances found in nutraceuticals has led to enhanced delivery and thereby improved activity [3,4,5]. Formulation into colloidal systems typically involves particles or matrix systems at sub-micrometer scales [6]. Various in vitro encapsulation techniques have been developed, including coacervation, liposome entrapment and spray drying, as comprehensively described in a recent review [7].

Among the more recent developments in encapsulation technology is the exploitation of the natural properties of plant cells, which can be used to produce microparticles based on cellular or subcellular sequestration, and nanoparticles based on protein assemblies. This approach, known as bioencapsulation, produces drug products already formulated in the DDV. The same term is used in agriculture and tissue engineering to describe living cells (such as bacteria or stem cells) incorporated into a protective matrix [8,9]. This review focuses on the bioencapsulation of protein drugs in plant cells and specifically considers the utilization of plant organelles and assemblies such as virus-like particles (VLPs) for the development of innovative DDVs. 

## 2. The Benefits of Encapsulation and Particulate Formulations

**Figure 1:**
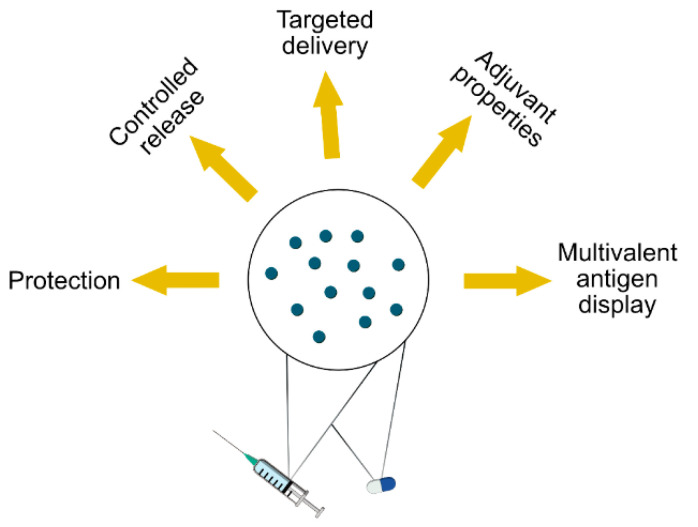
Benefits of encapsulating drugs into particles.

The increasing demand for drugs with new mechanisms of action has made drug delivery more challenging. Many of the most active small-molecule drugs are poorly water-soluble and therefore unfavorable for absorption. At the same time, advances in molecular biology have led to the explosive growth of biologics, including many drugs based on macromolecules such as antibodies, which are sensitive to enzymatic degradation and difficult to transport across biological barriers. This has prompted research into the development of new DDVs, including colloidal systems for the solubilization and controlled release of poorly water-soluble drugs, and particulate systems for the targeted delivery of proteins [6]. The advantages that can be achieved by encapsulating pharmaceuticals into particles are summarized in Figure 1. 

The encapsulation of drugs into nanoparticles, microparticles or polymer-based carriers protects the cargo from environmental effects. Oral drugs are usually administered as capsules or tablets, which are acceptable to most patients [1]. Shielding the active pharmaceutical ingredient from the environment not only protects the drug from the effects of stomach acid and digestive enzymes but also prevents off-target effects. However, although capsules and tablets are considered the gold standard of oral drug delivery, they only confer primary (macroscale) protection. For the precise control of drug release on a molecular scale, the active pharmaceutical ingredient needs to be encapsulated in additional layers such as nanoparticles that allow controlled diffusion into the environment, in some cases only following interaction with a specific target. This approach has already found clinical success by reducing dosing frequencies for certain drugs [1]. 

One of the best examples of the benefits of controlled delivery and release is chemotherapy, where drugs targeting a tumor show enhanced efficacy and pharmacokinetic behavior while preventing side effects [10]. By modifying the surface of nanocarriers with cell-specific ligands, drugs can be released directly in the vicinity of cancer cells, or even after the carrier is taken up by endocytosis, thus protecting healthy cells from exposure [11]. Strategies driven by receptor-mediated translocation are also used in vaccinology. Encapsulated antigens can be targeted to antigen-presenting cells (APCs) in a complex tissue (such as the intestinal lumen) using cell-penetrating peptides or tags like the cholera toxin B subunit (CTB). The latter binds to the GM1 ganglioside receptor expressed on intestinal epithelial and dendritic cells, ensuring that antigens fused to CTB are internalized and processed [12]. 

Particulate DDVs are also beneficial for vaccines because they possess inherent immunostimulatory properties. Accordingly, encapsulated antigens are taken up more efficiently by APCs than the corresponding soluble protein [13,14]. Furthermore, particulate protein assemblies display multiple copies of the antigen, typically in a regular array, which enhances the antibody response [13]. The benefits of particulate DDVs can be maximized by combining the antigen, a receptor-binding ligand and an adjuvant in one particle. Depending on efficacy, the carrier can even replace the adjuvant by providing the same biological function. This is because the beneficial effects of adjuvants are often derived from their particulate structure, which is common to many nanomaterials [14,15,16]. 

Encapsulation is particularly useful for the mucosal delivery of vaccines, which induces not only systemic immunity but also mucosal immunity, protecting mucosal barriers such as the intestine from invading pathogens. This is important because many of the most serious human pathogens enter the body via mucosal surfaces, leading to gastroenteric, genitourinary or respiratory diseases [17,18]. Similarly, many allergens enter the body via mucosal surfaces thus mucosal delivery is also the preferred route for allergen-specific immunotherapy and the induction of tolerance. Biocompatible polymers such as poly(lactic-co-glycolic acid), chitosan, silica and liposomes are commonly used as DDVs for this purpose, with chitosan favored due to its mucoadhesive properties [19,20].

The shift to non-invasive drug delivery strategies is not only desirable because patients find it more acceptable but also because it facilitates drug administration in farm animals and domestic pets to control diseases that pose an economic risk for farmers and/or the risk of zoonotic transmission to humans. Mucosal immunization, such as the administration of vaccines via drinking water or feed or (for aquatic species) via immersion, enables the vaccination of hundreds or thousands of animals over a short period of time without the effort required for injection and the stress caused to the vaccinated animals. Most licensed mucosal vaccines for veterinary applications are live-attenuated viruses for injection. Due to risks associated with those vaccines, there is an urgent need for new vaccine technologies to combat emerging zoonotic diseases more efficiently [21]. Vaccines based on particulate carriers may contribute to these new products, including the development of VLPs as mucosal vaccines against influenza A [22,23].

## 3. Plants as a Means to Achieve Bioencapsulation

Plants can facilitate bioencapsulation at the cellular and subcellular levels and also allow the synthesis of proteinaceous nanoparticles, thus providing many opportunities for the development of novel DDVs. Because of the high content of lignin and cellulose in the plant cell wall, plant cells are remarkably resistant to physical stress and enzymatic digestion. Although plants serve as the primary food source for many animals, the nutrients within plant cells can only be accessed with the help of commensal intestinal bacteria. The encapsulation of drugs and vaccines in plant cells therefore provides protection during passage through the upper digestive system but subsequently allows drug delivery to the intestinal lining. Cell-specific ligands fused to the encapsulated protein then promote uptake into intestinal epithelial cells and delivery to mucosal immune cells or across the endothelium into the circulation, promoting a systemic immune response and even allowing oral drugs to cross the blood-brain barrier [24]. 

**Figure 2:**
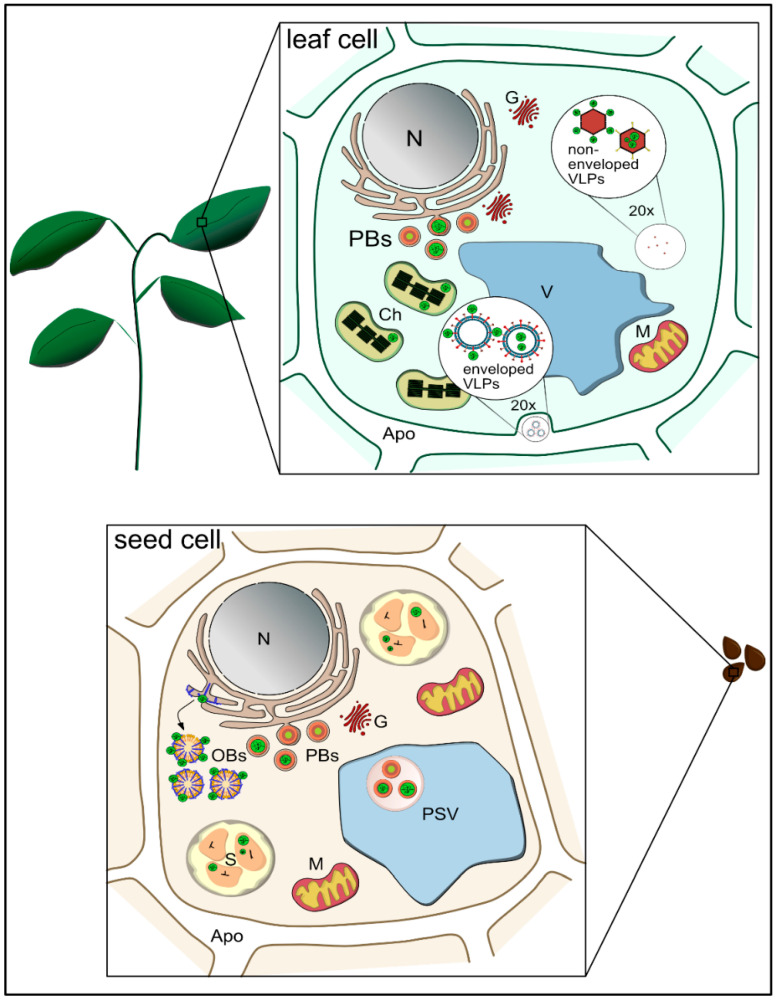
Plant-produced micro- and nanoparticles can be used to incorporate recombinant proteins (green circles). Specialized tissues in seeds (lower panel) are characterized by the presence of starch granules (S), oil bodies (OBs) or protein bodies (PBs), which may also be sequestrated into the protein storage vacuole (PSV). In leaves (upper panel), the formation of recombinant PBs can be induced ectopically, and plastid transformation enables the expression and accumulation of recombinant proteins in chloroplasts (Ch). In addition, nanoparticles such as enveloped and non-enveloped virus-like particles (VLPs) can be produced in planta. Recombinant proteins (green circles) may be incorporated into PBs or starch granules, associated to the surface of OBs, or they may be enclosed within or displayed on VLPs. N…Nucleus, V…Vacuole, G… Golgi, Apo… Apoplast.

Within the cell wall, plant cells feature a number of subcellular compartments that can provide an additional protective barrier. For example, storage organelles allow the stable intracellular accumulation of nutrients and energy reserves, including lipids, carbohydrates and proteins (Figure 2) [25]. Such organelles are mainly found in the cells of storage organs such as seeds and tubers, but they can be induced to form in other tissues by the overexpression of recombinant proteins, particularly those with structures that resemble native storage proteins. This endogenous encapsulation mechanism allows the long-term storage of recombinant proteins without degradation or loss of activity and offers a platform for the production of microparticles as DDVs.

Plant-derived polymers extracted from storage organelles can also be used as in vitro encapsulation materials. For example, zein is the major storage protein found in maize seeds and has been extensively studied due to its unique physicochemical and biological properties. It forms edible films that are tough, hydrophobic and resistant to microbial degradation, making them ideal as food and pharmaceutical coatings [26]. Zein nanoparticles are used for the in vitro encapsulation of sparingly-soluble molecules such as curcumin [27], aceclofenac [28], quercetin [29], and α-tocopherol [30]. 

Starch grains store high-energy carbohydrate resources in plants, and both the organelles and the starch polymers they contain have been developed as DDVs [31,32]. Starch polymers offer good biocompatibility and are therefore used in many different biomedical and pharmacological applications [33]. Even a potential adjuvant effect of starch microparticles was recently demonstrated [30]. 

Sporopollenin is a plant-based biopolymer known as “the diamond of the plant world” due to its extraordinary stability [34]. It is derived from plant spores and pollen, and when extracted it forms empty exines or microcapsules that can be loaded with enzymes [35], fish oils and drugs such as ibuprofen [36]. Sporopollenin not only shows remarkable physical and chemical resistance, it also has mucoadhesive properties and enhances the bioavailability of encapsulated molecules such as eicosapentaenoic acid from fish oil [34]. This has promoted interest in the development of sporopollenin microcarriers for oral drug and vaccine delivery [37,38].

One of the main advantages of using plant cells, organelles and plant-derived biopolymers for encapsulation is the ability to produce recombinant pharmaceutical proteins in plants and encapsulate them in vivo, without extraction and formulation. The production of recombinant proteins in plants (molecular farming) began in the 1990s following the assembly of functional monoclonal antibodies in tobacco leaves [39] and the expression of human serum albumin in tobacco and potato plants and cell cultures [40]. The first combined use of plants as an expression host and DDV involved the expression of vaccine antigens in potato tubers [41]. Raw tuber was administered to mice (and later humans) in a series of preclinical and clinical trials against bacterial diarrhea, hepatitis and norovirus [42,43]. This approach was proposed as a strategy to facilitate vaccination in developing countries by eliminating the reliance on sterile injections and allowing the source of vaccines to be grown locally, thus removing the need for a cold chain. However, one of the drawbacks of plant tissues expressing recombinant proteins for oral vaccination is the variable dose. It is now recognized that some form of minimal processing (such as lyophilization) is necessary to evaluate quality attributes such as antigen concentration in order to ensure standardized doses. Even so, the plant cell wall survives lyophilization and continues to protect the encapsulated recombinant protein, which remains properly folded and active following rehydration even after storage at ambient temperatures for more than 2 years [44,45,46]. 

The early years of molecular farming saw the exploration of many alternative platforms, but the community has now consolidated around a small number of well-characterized systems that make it easier to apply the principles of pharmaceutical good manufacturing practice (GMP). The principal systems are transgenic plants (typically tobacco, cereals and fruit/vegetable crops), transient expression in tobacco, and plant cell suspension cultures, allowing competition with traditional platforms based on microbial and mammalian cells [47]. Stable expression involves the integration of DNA into the plant genome, resulting in transgenic plants/cell lines when the DNA integrates into the nucleus, or transplastomic plants/cell lines if DNA integrates into the plastids. Nuclear transformation is more widely practiced because this approach works in many species, and the resulting proteins can be directed to the secretory pathway or other subcellular compartments for post-translational modification (PTM). In contrast, plastid transformation causes the recombinant protein to accumulate directly in the plastid and PTM is not possible. The advantages of plastid transformation are the enhanced containment (the plastid genome of most crops is maternally inherited, minimizing the risk of gene transfer by outcrossing) and the high protein yields, because there may be up to 10,000 copies of the plastid genome in leaf cells [48,49]. Several therapeutic proteins have been produced in transplastomic plants, including ACE-2/Ang(1-7) [44], pro-IGF1 [50] and vaccine antigens against polio [12,51], dengue [52], tetanus toxin [53] and tuberculosis [54].

Whereas transgenic and transplastomic plants/cells are stable resources providing a permanent, scalable platform for recombinant protein production, transient expression systems involve the short-term expression of proteins in the leaves of plants infiltrated with genetically modified bacteria or infected with recombinant viruses. Transient expression is much faster than stable transformation and is ideal for urgent responses to emerging epidemic or pandemic diseases. Large-scale facilities to manufacture vaccines by transient expression in *Nicotiana benthamiana* have been established by companies such as Kentucky Bioprocessing (Owensboro, KT, USA), iBio (Bryan, TX, USA) and Medicago Inc. (Quebec, QC, Canada), the latter producing VLP-based vaccines against seasonal and pandemic influenza strains [55] and also against COVID-19 [56]. Plants have therefore emerged as a scalable, safe, sustainable and cost-effective platform for the rapid production of vaccines and drugs to address new pandemic diseases [57]. The ability to assemble particulate structures such as VLPs provides opportunities for the production of low-cost vaccines, a necessity for developing countries [58] particularly when minimally processed edible plant tissues are administered via the mucosal route [59,60,61]. 

## 4. Plant-Derived Microparticles: Storage Organelles for Bioencapsulation 

Seeds have evolved an extraordinary capacity to accumulate nutrients and energy reserves within specialized tissues, thus providing resources for the germinating embryo even after years of storage. Seeds can store protein reserves in protein bodies or storage vacuoles, lipids in oil bodies, and carbohydrates in starch granules. This native encapsulation strategy can be exploited to stockpile recombinant proteins in a stable environment that prevents proteolytic degradation. In molecular farming, this strategy is used to enhance the yields of recombinant proteins and for drug delivery. The latter is discussed in more detail below, and examples are listed in Table 1. 

### 4.1. Encapsulation in Protein Bodies

Cereal and legume seeds contain a large number of storage compartments for proteins, and this is the typical destination of recombinant proteins expressed in seeds. The two main types of protein storage compartments are known as protein bodies, which are derived directly from the membrane of the endoplasmic reticulum (ER), and protein storage vacuoles, which can be reached via Golgi-independent or Golgi-dependent pathways [70]. The deposition of recombinant proteins within such organelles extends the basic protection of the plant cell wall by providing an extra membrane barrier, and further protection against proteolysis is conferred by the dense packing of the protein [71,72]. 

The protection offered by protein bodies is useful for oral drug administration because the DDV can better resist the harsh conditions of the gastrointestinal tract. For example, allergen-specific immunotherapy requires the regular administration of antigens over a long period, so oral administration is more convenient than injection. However, the oral administration of crude allergen extracts requires up to 100-fold higher doses than injected allergens due to premature degradation in the gut [62,73]. To overcome this drawback, transgenic rice seeds were used for the production and delivery of T-cell epitopes corresponding to various allergenic proteins, such as Japanese cedar pollen or dust mite allergens [74]. By targeting cedar pollen allergens to protein storage organelles such as protein bodies (PB-I in rice) and protein storage vacuoles (PB-II in rice), the encapsulated allergens were protected against proteolytic digestion following oral delivery in mice. The immunostimulatory peptides were delivered to the lymphoid tissue in the gut and taken up by immune cells, leading to the significant suppression of allergen-specific IgE antibodies. Interestingly, in vitro digestion assays showed that the antigens were more stable in PB-I than PB-II, with the latter requiring a three-fold higher dose to achieve the same efficacy as the antigen encapsulated in PB-I [74]. 

Small proteins have also been stably incorporated into protein bodies in rice endosperm. Griffithsin is a 12.7-kDa algal lectin that significantly inhibits the ability of several viruses (including HIV) to enter target cells by binding selectively to mannose-rich glycans on viral glycoproteins. The encapsulation of griffithsin preserved its activity in crude endosperm extracts, which were shown to inhibit HIV entry in cell lines [59]. This provides an opportunity to produce inexpensive topical microbicides for the prevention of HIV, based on the preparation of crude extracts from transgenic rice seeds.

The mechanism of protein body biogenesis and the sequestration mechanism in cereals have been extensively studied [75,76,77,78,79]. Although the process is not fully understood, some aspects have been characterized in sufficient detail to induce ectopic protein bodies in non-storage tissues such as leaves. The maize storage protein γ-zein has gained particular recognition because it can induce protein body formation even when the other zeins are absent. A truncated version of γ-zein, corresponding to the first 112 N-terminal amino acids including a 19-kDa signal peptide, was found to be sufficient to induce protein body formation. Ectopic protein storage bodies have been induced not only in the vegetative tissues of plants but also in fungi and mammalian cells [80,81].

The formation of ectopic protein bodies opened up new approaches for the bioencapsulation of recombinant proteins by fusing a target protein to the γ-zein N-terminal fragment as a protein body-inducing tag, commercially developed as the Zera tag by Era Biotech [82,83,84]. Pharmaceutically relevant proteins such as calcitonin, human epidermal growth factor (hEGF) and human growth hormone were among the first targeted to ectopic protein bodies in *Nicotiana benthamiana* leaves [81]. Although these artificial protein bodies are structurally dissimilar to those naturally produced in maize, which feature multiple layers of different zeins [85], they share similar properties. The spherical, membrane-bound particles are ~1 µm in diameter with a density of ~1.20 g/cm³ (determined for protein bodies containing green fluorescent protein (GFP)), which facilitates downstream processing. Interactions with chaperones, as seen in native protein bodies, encourage efficient protein folding [81]. 

Zein protein bodies not only accumulate and protect recombinant proteins, they also act as an adjuvant when injected into mice, providing an ideal strategy to deliver vaccines. This became evident when the immune response to antigens incorporated into protein bodies could not be enhanced by adding Freund’s adjuvant [64]. This supports earlier work showing that empty protein bodies administered with the soluble antigen enhanced the immune response compared to the antigen alone [86]. 

**Figure 3:**
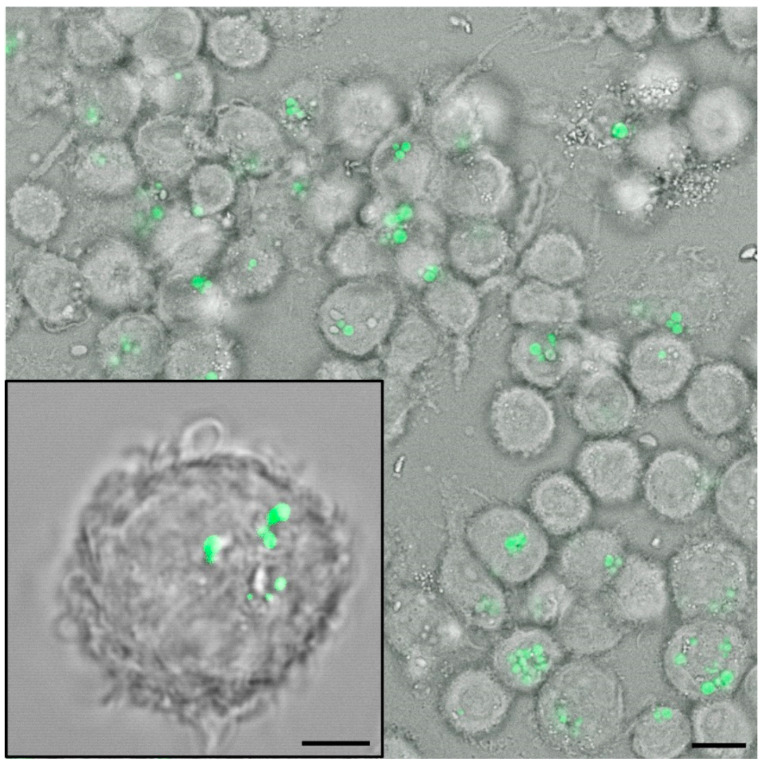
Uptake of zein-green fluorescent protein (GFP) bodies into antigen presenting cells, as described in Schwestka et al. 2020 [89]. Upon 2 h of incubation, zein-GFP protein bodies (green) are taken up by U937 cells. Inset shows an enlarged confocal image of a cell. Bars represent 10 µm.

Another important characteristic of zein protein bodies is their ability to interact with cell membranes, reflecting the presence of proline-rich repeats in the γ-zein polypeptide. The repetitive domain (VHLPPP)_8_ was linked to a cell-penetrating peptide, suggesting zein-protein bodies are taken up efficiently by cells [87,88]. Our recent in vitro work has also revealed that zein-GFP protein bodies are taken up more efficiently than synthetic polystyrene particles of the same size when administered to intestinal epithelial cells (Figure 3) [89]. 

However, the induction of artificial protein bodies is not always successful when the zein sequence is used as a fusion tag. The ectodomain of influenza hemagglutinin is, to our knowledge, the largest fusion partner that has been incorporated into artificial protein bodies [64]. Other viral antigens, such as the HIV negative factor (Nef) and CAP256 gp140 envelope antigen, were not incorporated into protein bodies even though the recombinant protein accumulated in the ER, and subsequent immunization was successful [90]. It is unclear why ectopic protein body formation was inhibited by these proteins, and understanding (and overcoming) these limitations would make the strategy feasible for a wider range of proteins. 

The large-scale production of protein bodies requires an effective purification method. Currently, this is usually achieved by gradient ultracentrifugation, which is a barrier to commercial development [65,86]. We recently established an alternative procedure based on serial filtration, which is much more scalable. However, the two consecutive tangential flow filtration steps only concentrate particles of a specific size, thus achieving a protein body purity of 66.5% with the remainder being host cell debris [89]. As a result, future applications must focus on drug delivery strategies such as oral delivery that do not require extensive purification and sterile conditions. Such vaccine formulations may even benefit from the immunostimulatory properties of plant subcellular debris such as starch particles [32,86,87].

### 4.2. Oleosin-Targeted Deposition

Many seeds are rich in lipids, mainly triacylglycerols, which are stored in organelles known as oil bodies. These are 0.5–2.5 µm in diameter enclosed within a phospholipid layer that is densely covered with at least three types of protein: steroleosin, caleosin and oleosin. The latter is the most abundant and gives rise to the most intriguing properties of oil bodies, shielding the underlying phospholipids and thus avoiding aggregation and coalescence. Oil bodies are not only remarkably stable in planta but also remain as discrete particles after extraction and long-term storage [91,92,93].

Recombinant proteins can be targeted to the surface of oil bodies by expressing them as fusions with oleosin. The oleosin fusion protein is transported through the ER to the oil bodies in the cytosol, and the recombinant protein accumulates to high levels on the surface [93,94]. The lipophilic nature of oil bodies makes them easy to separate from the aqueous extraction medium by floating centrifugation, which simplifies downstream processing [95]. This technology has enabled the accumulation of proteins such as β-glucuronidase (the first to be reported), xylanase and hirudin in *Brassica napus* seeds [95,96,97], insulin, human fibroblast growth factor 9 (hFGF-9) and hEGF in *Arabidopsis thaliana* seeds [98,99,100], and hFGF-9, hEGF and antimicrobial peptides in safflower seeds [66,67,101,102]. All of these proteins remained functional in vitro. The emulsifying properties of oil bodies in safflower seeds were shown to promote absorption when topically applied to the skin of wounded rodents [66]. The transdermal drug delivery of oil body-linked hEGF significantly accelerated wound healing and tissue regeneration, and the mechanism may be similar to that seen with drugs delivered via liposomes [66]. 

Growth factors such as FGF and EGF were able to promote cell proliferation even when bound to oil bodies [96,97,99]. However only a few of the many proteins expressed as oleosin fusions remain active as part of the oil body and most need to be cleaved from their oleosin fusion partner. This requires the incorporation of a protease cleavage site at the fusion protein junction. Following cleavage, in all studies reported thus far, the released protein retained full activity and did not require refolding. For example, the anticoagulant hirudin was inactive in the oil body but regained its function after cleavage [95] showing that it must have retained the three disulfide bonds required for full activity [103]. Other PTMs have not been detected on oleosin-fusion proteins. For example, xylanase expressed as an oleosin fusion lacked the N-linked glycans found on the native protein, but these are not required for its activity [97]. Similarly, the N-glycans normally found on human growth factors such as hFGF-9 were also missing from the oleosin fusion protein [66]. Oleosin fusion technology is therefore unsuitable for proteins that require complex PTMs for activity. Other limitations, such as the size and biochemical properties of proteins needed to form stable particles, remain to be determined. 

The use of recombinant oil bodies as a platform for antigen display has not been reported thus far. However, this approach offers potential advantages such as oral administration following limited purification based on established protocols [95], stability when isolated and stored at room temperature, and resistance to digestion in the stomach. The tight packing of oleosins on the surface of oil bodies and the presence of pepsin-resistant domains seem to confer protection, which would be enhanced even further if encapsulated by the cell wall [104]. It is unclear whether antigens displayed on the surface would be taken up by APCs to elicit a protective immune response. 

Oil body formation is not entirely restricted to seeds, although they show the strongest potential given the abundance of lipids in these organs. Oleosin-GFP fusion proteins were successfully targeted to bona fide oil bodies in *N. benthamiana* leaves, which were remarkably similar to the oil bodies in embryos. However, leafy tissues cannot compete with the lipid metabolism in oilseeds—the number of recovered oil bodies was therefore very low, and they were prone to aggregation in planta [93]. For commercial development, oily seed crops amenable to genetic transformation are ideal. SemBioSys Bio-Pharmaceutical established a platform based on the oleosin-fusion technology in safflower seeds and tested several products in clinical trials. 

### 4.3. Deposition in Starch Granules

Starch is the main storage carbohydrate in higher plants. Extensive research focusing on the regulation of starch biosynthesis in plants has facilitated the bioengineering of starch synthesis, improving the nutritional quality of food crops and producing starch with modified physicochemical properties for industrial applications [105,106]. Starch is deposited in starch granules that form within organelles known as amyloplasts. The enzymes required for starch biosynthesis (e.g., starch synthase and starch branching enzyme) are found within the amyloplasts associated with starch granules. Interestingly, the binding of proteins to the polysaccharide matrix confers both stability and resistance against protease degradation, even after extraction [107]. Starch granules have been used for the encapsulation of malaria vaccines by attaching antigens from the parasite *Plasmodium falciparum* to the enzyme granule-bound starch synthase in algae. Immunization of mice by the intraperitoneal and oral administration of starch particles together with an adjuvant conferred protection against *P. falciparum* [69]. Furthermore, the heat labile enterotoxin B subunit (LT-B) of *Escherichia coli* was unintentionally deposited into the starch granules of transgenic maize, probably reflecting the presence of intrinsic amyloplast targeting signals [68]. Although starch-based antigens can induce an oral immune response in mice and the potent oral immunogen LT-B appears preferentially targeted to starch granules, follow-up studies have yet to be reported. Current research focuses mainly on the use of starch for in vitro encapsulation because it is an inexpensive, biocompatible polymer. However, amyloplast targeting allows the encapsulation process to occur in planta at the same time as protein expression, making extensive in vitro formulation procedures obsolete. Edible plant tissues containing starch grains would allow the oral administration of minimally processed vaccines, significantly reducing the costs of production and administration.

## 5. Plant-Derived Nanoparticles

In 1882, a “filterable infectious agent” was defined as the cause of tobacco mosaic disease in plants, marking the first research into viruses and VLPs [108]. Almost four decades later, electron microscopy enabled the visualization of tobacco mosaic virus as rod-shaped structures [109]. Since then, more than 5000 different viruses have been identified that infect all forms of life. Researchers are not only interested in their status as pathogens, but also in the unique physicochemical properties that make them suitable for medical applications, such as their remarkable stability, their nanoscale structures and their ability to assemble spontaneously from their components [110,111]. VLPs are particularly useful as vaccines because they resemble the structure of the genuine virus but lack the nucleic acid and therefore provoke an immune response without causing infection. VLPs also possess inherent immunostimulatory properties because they are particulate structures, and this can be exploited in immunotherapy [112]. A summary of the most recent studies, carried out with plant-produced VLPs, is presented in Table 2.

### 5.1. Animal-Derived VLPs Expressed in Plants 

Until recently, most studies on the use of VLPs as vaccines have focused on animal viruses because these are the agents that cause disease in humans [113,114]. The first commercially available recombinant vaccine was a VLP based on hepatitis B virus produced in yeast, and it was approved in 1986 [113,115]. This was followed by the approval of VLP vaccines based on human papilloma virus (HPV) produced in yeast and insect cells [116]. As highlighted elsewhere in this issue [117], the use of fermenter-based expression platforms is expensive and the vaccines are largely inaccessible in developing countries, but plants offer an inexpensive and more scalable alternative [47,118]. Indeed, the hepatitis B surface antigen (HBsAg) was among the first recombinant proteins expressed in transgenic tobacco plants and was also shown to form VLPs that elicited humoral and cellular immune responses in mice, similar to those obtained with a commercial vaccine [119]. Since then, many other virus structural proteins have been expressed in plants, including foot and mouth disease coat protein and the HPV L1 coat protein [58,120,121,122].

**Table 2:** Most recent examples of in planta produced virus-like particles (VLPs).

Virus-Like Particles	Expression System	Size	In Vivo Studies	Ref.
Enveloped	Tobacco	0.05–0.150	Immunization against H5/H1	[123]
	Tobacco	0.025–0.04	Immunization against dengue viral protein	[124,125]
	Tobacco	~0.1	Immunization against SARS-CoV-2	[56]
Non-enveloped	Cowpea	0.030	Adjuvant in anti-cancer vaccines	[126,127]
	Tobacco	0.07	Immunization against African horse sickness	[128,129]
	Tobacco	0.016	Immunization against PCV-2	[130]
	Tobacco	0.025–0.039	Immunization against various HPV types	[117]
	Tobacco	0.025–0.03	Immunization against VNN	[131]
	Tobacco	~0.03	Enhanced immunogenicity of ZE3 antigen via RIC vaccine platform	[132]
	Tobacco	0.025–0.03	Immunization against WNV	[133]

Abbreviations: Ref.: references; H5: hemagglutinin subtype 5; PCV-2: porcine circovirus type 2; HPV: human papilloma virus; VNN: viral nervous necrosis; ZE3: Zika envelope domain III; RIC: recombinant immune complex; WNV: West Nile virus.

Following the successful production of VLPs based on HBsAg, more complex particles were produced containing more than one type of protein subunit, and even enveloped particles have now been expressed in plants [58,122,125,134,135,136]. The hepatitis B core antigen (HbcAg) has been expressed not only as a potential VLP-based vaccine against hepatitis B virus but also as a carrier for other vaccines due to its strong inherent immunogenicity [134]. For example, in a recent study using *N. benthamiana,* the high-level production and immunogenicity of HBcAg-based VLPs presenting a West Nile virus antigen has been demonstrated [133]. An important development was the generation of VLPs containing tandem core HBcAgs dimers, which enables the display of full proteins such as GFP or nanobodies in one of the major insertion regions within one of the two tandem HBcAg copies [135]. Further improvements were achieved by the use of the SpyTag/SpyCatcher conjugation system, which exploits the formation of a strong isopeptide bond within the *Streptococcus pyogenes* FbaB protein CnaB2 domain. If the tandem HBcAg core particles carry the SpyCatcher sequence (a 12.3-kDa portion of the CnaB2 domain), then any protein carrying the 13-amino-acid SpyTag can form an irreversible isopeptide bond. This has been used, for example, to conjugate the HIV antigen p24 to HBcAg VLPs in planta [136]. 

One of the most promising VLP-based vaccine candidates is Medicago’s quadrivalent VLP influenza vaccine, which recently completed phase III clinical trials [55]. The influenza hemagglutinin protein can form enveloped VLPs in planta by budding from the plasma membrane, independent of any carrier protein [123]. The quadrivalent vaccine was therefore produced by co-expressing hemagglutinins from different viral strains, resulting in the formation of heterologous VLPs carrying a mixture of antigens [137]. The vaccines can be produced in plants in a matter of weeks, compared to 6 months or more for the conventional vaccine produced in chicken eggs, also overcoming the risk of reinfection and the unsuitability of egg-based vaccines for recipients with egg allergies. The same approach is now being applied to SARS-CoV-2 in an attempt to develop vaccines against COVID-19. Just 20 days after receiving the SARS-CoV-2 gene sequence, Medicago successfully produced VLPs that are currently undergoing phase III testing [56]. 

### 5.2. Plant-Derived VLPs and Viral Nanoparticles

Although plants can synthesize VLPs based on animal viruses, all such products carry a residual risk of infection because they could theoretically accommodate viral nucleic acid present in the vaccine recipient. In contrast, plant viruses cannot replicate in animals and therefore can be used safely as vaccines or DDVs even if the original viral genome is present. Plant viruses can therefore be developed as either VLPs (resembling the virus but lacking the genome) or so-called viral nanoparticles (VNPs) with the genome intact. The advantage of VNPs is that they still replicate in plants, allowing large quantities to be produced naturally, but they can still be used as DDVs or nanoscale scaffolds for the display of antigens, thus functioning as recombinant vaccines [138]. One of the first examples of plant-derived VNPs was cowpea mosaic virus (CPMV), which can produce high yields when inoculated onto *N. benthamiana* even though this is not its natural host [139]. The expression of CPMV coat proteins can also be used to produce VLPs [110]. Both VLPs and VNPs can be engineered to display external peptides (such as vaccine antigens or targeting peptides for drug delivery) but the VLPs are advantageous as DDVs because the empty capsid can be loaded with drugs, fluorophores or contrast agents, allowing their development as therapeutic, diagnostic or even theranostic reagents [138,140,141,142]. The medical applications of CPMV are facilitated by its inherent ability to interact with vimentin, an intermediate filament protein present on all mesenchymal cells but upregulated in certain tumors, allowing the targeted delivery of drugs and imaging reagents to breast and prostate tumors [143,144]. VLPs are usually administered by injection but CPMV-derived VLPs were recently shown to be robust and stable in a simulated gastrointestinal environment in vitro and in the presence of porcine gastrointestinal fluids [145]. This suggests that CPMV may be suitable as an oral DDV. 

## 6. Remaining Challenges 

As elaborated in this review, it has been demonstrated that plants are suitable expression systems for a variety of particulate structures. Despite the high potential of plant-derived particulate structures, the corresponding products under development have yet to reach the market. One of the most challenging technical aspects concerns downstream processing. The separation of the desired particles from the plant extract often requires extensive clarification which renders the process cost-intensive. For the isolation of PBs and VLPs, separation by density gradient ultracentrifugation has become the method of choice. However, this requires expensive equipment and time-intensive centrifugation and fractionation steps. For upscaled production, alternative purification strategies such as tangential-flow filtration or depth filtration have to be established [129]. Despite this challenge, Medicago Inc. produces influenza and SARS-CoV-2 VLPs at industrial scales with high purity and a favorable safety profile [55,56].

## 7. Conclusions and Perspectives

Plant-based expression platforms allow the direct bioencapsulation of recombinant proteins during the manufacturing process, which could facilitate the development of new DDVs. Natural plant-derived polymers such as storage proteins and starch have long been used to encapsulate drugs in vitro but can also be exploited directly within the production host. Recombinant proteins accumulating inside plant cells are naturally encapsulated by the cell wall, and additional, subcellular levels of protection can be achieved by exploiting natural storage organelles for proteins, lipid and carbohydrates, or inducing the formation of these structures in other tissues, including leaves. In addition to the microparticles formed by encapsulation in cells and organelles, nanoparticles can be produced by expressing virus components that spontaneously assemble into VLPs. These can be derived from animal or plant viruses, providing ample scope for the development of novel vaccines based on self-assembling animal virus proteins or chimeric systems in which animal virus epitopes are displayed on a plant virus scaffold. VLPs are among the most promising plant-derived pharmaceutical products because they can display heterologous peptides and carry an internal cargo of drugs or imaging molecules, making them extremely versatile. By successfully completing the clinical phase III, the quadrivalent VLP influenza vaccine candidate of Medicago Inc. is close to entering the market, and this would boost the acceptance of plant-derived pharmaceuticals and open avenues also for other companies. The current COVID-19 pandemic has once more highlighted the need for alternative expression platforms to satisfy the huge demand for diagnostic, therapeutic and prophylactic reagents. Similar to its VLP-based vaccine candidate against influenza, Medicago Inc. have recently entered clinical phase III studies with their VLP-based vaccine candidate against SARS-CoV-2. Moreover, iBio have announced to focus on the production of VLP-based vaccines produced in *N. benthamiana.* The diverse particulate or encapsulated formulations that can be manufactured in plants provide a broad range of strategies for drug and vaccine delivery by injection but also via oral and other mucosal routes for the prevention and treatment of enteral or respiratory diseases. The latter routes of administration can certainly help to improve the reach of vaccination campaigns and thereby contribute to disease management in the future.

## Figures and Tables

**Table 1:** Selected examples and comparison of in planta produced microparticles.

Particles	Expression System	Size [µm]	PTMs	In Vivo Studies	Ref.
Protein bodies	Rice	~1	+	ASIT against Japanese Cedar pollen allergen	[62,63]
	Tobacco	1–2	+	Immunization against H5	[64]
	Tobacco	0.6–1	n.d.	Immunization against BTV serotypes	[65]
Oil bodies	Safflower seeds	0.5–2.5	-	Transdermal drug delivery of hormones: rhFGF9, hEGF	[66,67]
Starch granules	Maize	~2	-	n.a.; only in vitro digestion of encapsulated LT-B antigen	[68]
	Algae	~1.5	-	Immunization against plasmodial antigens	[69]

Abbreviations: PTMs: posttranslational modifications; Ref.: references; ASIT: allergen-specific immunotherapy; n.d.: not determined; H5: hemagglutinin subtype 5; BTV: bluetongue mosaic virus; rhFGF9: recombinant human fibroblast growth factor-9; hEGF: human epidermal growth factor; n.a.: not available.

## Data Availability

Not applicable.

## References

[1] Anselmo A.C., Gokarn Y., Mitragotri S. (2019). Non-invasive delivery strategies for biologics. Nat. Rev. Drug Discov..

[2] Sahni N., Cheng Y., Middaugh C.R., Volkin D.B., Wang B., Hu L., Siahaan T.J. (2016). Vaccine Delivery. Drug Delivery.

[3] Jones D., Caballero S., Davidov-Pardo G. (2019). Bioavailability of nanotechnology-based bioactives and nutraceuticals. Adv. Food Nutr. Res..

[4] Yeung A.W.K., Souto E.B., Durazzo A., Lucarini M., Novellino E., Tewari D., Wang D., Atanasov A.G., Santini A. (2020). Big impact of nanoparticles: Analysis of the most cited nanopharmaceuticals and nanonutraceuticals research. Curr. Res. Biotechnol..

[5] Souto E.B., Zielinska A., Souto S.B., Durazzo A., Lucarini M., Santini A., Silva A.M., Atanasov A.G., Marques C., Andrade L.N. (2020). (+)-Limonene 1,2-Epoxide-Loaded SLNs: Evaluation of Drug Release, Antioxidant Activity, and Cytotoxicity in an HaCaT Cell Line. Int. J. Mol. Sci..

[6] Boyd B.J. (2008). Past and future evolution in colloidal drug delivery systems. Expert Opin. Drug Deliv..

[7] Perry S.L., McClements D.J. (2020). Recent advances in encapsulation, protection, and oral delivery of bioactive proteins and peptides using colloidal systems. Molecules.

[8] John R.P., Tyagi R.D., Brar S.K., Surampalli R.Y., Prevost D. (2011). Bio-encapsulation of microbial cells for targeted agricultural delivery. Crit. Rev. Biotechnol..

[9] Majewski R.L., Zhang W., Ma X., Cui Z., Ren W., Markel D.C. (2016). Bioencapsulation technologies in tissue engineering. J. Appl. Biomater. Funct. Mater..

[10] Raave R., van Kuppevelt T.H., Daamen W.F. (2018). Chemotherapeutic drug delivery by tumoral extracellular matrix targeting. J. Control. Release.

[11] Sutradhar K.B., Amin M.L. (2014). Nanotechnology in Cancer Drug Delivery and Selective Targeting. ISRN Nanotechnol..

[12] Daniell H., Kulis M., Herzog R.W. (2019). Plant cell-made protein antigens for induction of Oral tolerance. Biotechnol. Adv..

[13] Snapper C.M. (2018). Distinct Immunologic Properties of Soluble Versus Particulate Antigens. Front. Immunol..

[14] Zhu M., Wang R., Nie G. (2014). Applications of nanomaterials as vaccine adjuvants. Hum. Vaccines Immunother..

[15] Jiao Q., Li L., Mu Q., Zhang Q. (2014). Immunomodulation of nanoparticles in nanomedicine applications. Biomed. Res. Int..

[16] Torres F.G., Troncoso O.P., Pisani A., Gatto F., Bardi G. (2019). Natural Polysaccharide Nanomaterials: An Overview of Their Immunological Properties. Int. J. Mol. Sci..

[17] Kim S.H., Jang Y.S. (2017). The development of mucosal vaccines for both mucosal and systemic immune induction and the roles played by adjuvants. Clin. Exp. Vaccine Res..

[18] Zhu Q., Berzofsky J.A. (2013). Oral vaccines: Directed safe passage to the front line of defense. Gut Microbes.

[19] Shim S., Yoo H.S. (2020). The Application of Mucoadhesive Chitosan Nanoparticles in Nasal Drug Delivery. Mar. Drugs.

[20] Lima I.A., Khalil N.M., Tominaga T.T., Lechanteur A., Sarmento B., Mainardes R.M. (2018). Mucoadhesive chitosan-coated PLGA nanoparticles for oral delivery of ferulic acid. Artif. Cells Nanomed. Biotechnol..

[21] Topp E., Irwin R., McAllister T., Lessard M., Joensuu J.J., Kolotilin I., Conrad U., Stoger E., Mor T., Warzecha H. (2016). The case for plant-made veterinary immunotherapeutics. Biotechnol. Adv..

[22] Mohan T., Berman Z., Luo Y., Wang C., Wang S., Compans R.W., Wang B.Z. (2017). Chimeric virus-like particles containing influenza HA antigen and GPI-CCL28 induce long-lasting mucosal immunity against H3N2 viruses. Sci. Rep..

[23] Vacher G., Kaeser M.D., Moser C., Gurny R., Borchard G. (2013). Recent advances in mucosal immunization using virus-like particles. Mol. Pharm..

[24] Kwon K.C., Daniell H. (2016). Oral Delivery of Protein Drugs Bioencapsulated in Plant Cells. Mol. Ther..

[25] Stoger E., Ma J.K., Fischer R., Christou P. (2005). Sowing the seeds of success: Pharmaceutical proteins from plants. Curr. Opin. Biotechnol..

[26] Shukla R., Cheryan M. (2001). Zein: The industrial protein from corn. Ind. Crop. Prod..

[27] Patel A., Hu Y., Tiwari J.K., Velikov K.P. (2010). Synthesis and characterisation of zein–curcumin colloidal particles. Soft Matter.

[28] Karthikeyan K., Vijayalakshmi E., Korrapati P.S. (2014). Selective interactions of zein microspheres with different class of drugs: An in vitro and in silico analysis. AAPS PharmSciTech.

[29] Penalva R., Gonzalez-Navarro C.J., Gamazo C., Esparza I., Irache J.M. (2017). Zein nanoparticles for oral delivery of quercetin: Pharmacokinetic studies and preventive anti-inflammatory effects in a mouse model of endotoxemia. Nanomedicine.

[30] Luo Y., Zhang B., Whent M., Yu L.L., Wang Q. (2011). Preparation and characterization of zein/chitosan complex for encapsulation of alpha-tocopherol, and its in vitro controlled release study. Colloids Surf. B Biointerfaces.

[31] Troncoso O.P., Torres F.G. (2020). Non-conventional starch nanoparticles for drug delivery applications. Med. Devices Sens..

[32] Moreno-Mendieta S., Barrios-Payan J., Mata-Espinosa D., Sanchez S., Hernandez-Pando R., Rodriguez-Sanoja R. (2017). Raw starch microparticles have immunostimulant activity in mice vaccinated with BCG and challenged with Mycobacterium tuberculosis. Vaccine.

[33] Qiu C., Wang C., Gong C., McClements D.J., Jin Z., Wang J. (2020). Advances in research on preparation, characterization, interaction with proteins, digestion and delivery systems of starch-based nanoparticles. Int. J. Biol. Macromol..

[34] Mackenzie G., Boa A.N., Diego-Taboada A., Atkin S.L., Sathyapalan T. (2015). Sporopollenin, The Least Known Yet Toughest Natural Biopolymer. Front. Mater..

[35] Yilmaz E. (2012). Enantioselective enzymatic hydrolysis of racemic drugs by encapsulation in sol-gel magnetic sporopollenin. Bioprocess. Biosyst. Eng..

[36] Diego-Taboada A., Cousson P., Raynaud E., Huang Y., Lorch M., Binks B.P., Queneau Y., Boa A.N., Atkin S.L., Beckett S.T. (2012). Sequestration of edible oil from emulsions using new single and double layered microcapsules from plant spores. J. Mater. Chem..

[37] Lale S.V., Gill H.S. (2018). Pollen grains as a novel microcarrier for oral delivery of proteins. Int. J. Pharm..

[38] Uddin M.J., Gill H.S. (2017). Ragweed pollen as an oral vaccine delivery system: Mechanistic insights. J. Control. Release.

[39] Hiatt A., Caffferkey R., Bowdish K. (1989). Production of antibodies in transgenic plants. Nature.

[40] Sijmons P., Dekker B., Schrammeijer B., Verwoerd T., van den Elzen P., Hoekema A. (1990). Production of correctly processed human serum albumin in transgenic plants. Biotechnology.

[41] Haq T., Mason H., Clements J., Arntzen C.J. (1995). Oral immunization with a recombinant bacterial antigen produced in transgenic plants. Science.

[42] Tacket C., Mason H., Losonsky G., Clements J.D., Levine M.M., Arntzen C.J. (1998). Immunogenicity in humans of a recombinant bacterial antigen delivered in a transgenic potato. Nat. Med..

[43] Richter L.J., Thanavala Y., Arntzen C.J., Mason H.S. (2000). Production of hepatitis B surface antigen in transgenic plants for oral immunization. Nat. Biotechnol..

[44] Daniell H., Mangu V., Yakubov B., Park J., Habibi P., Shi Y., Gonnella P.A., Fisher A., Cook T., Zeng L. (2020). Investigational new drug enabling angiotensin oral-delivery studies to attenuate pulmonary hypertension. Biomaterials.

[45] Herzog R.W., Nichols T.C., Su J., Zhang B., Sherman A., Merricks E.P., Raymer R., Perrin G.Q., Hager M., Wiinberg B. (2017). Oral Tolerance Induction in Hemophilia B Dogs Fed with Transplastomic Lettuce. Mol. Ther..

[46] Su J., Zhu L., Sherman A., Wang X., Lin S., Kamesh A., Norikane J.H., Streatfield S.J., Herzog R.W., Daniell H. (2015). Low cost industrial production of coagulation factor IX bioencapsulated in lettuce cells for oral tolerance induction in hemophilia B. Biomaterials.

[47] Fischer R., Buyel J.F. (2020). Molecular farming—The slope of enlightenment. Biotechnol. Adv..

[48] Bock R. (2001). Transgenic plastids in basic research and plant biotechnology. J. Mol. Biol..

[49] Daniell H., Chan H.T., Pasoreck E.K. (2016). Vaccination via Chloroplast Genetics: Affordable Protein Drugs for the Prevention and Treatment of Inherited or Infectious Human Diseases. Annu. Rev. Genet..

[50] Park J., Yan G., Kwon K.C., Liu M., Gonnella P.A., Yang S., Daniell H. (2020). Oral delivery of novel human IGF-1 bioencapsulated in lettuce cells promotes musculoskeletal cell proliferation, differentiation and diabetic fracture healing. Biomaterials.

[51] Daniell H., Rai V., Xiao Y. (2019). Cold chain and virus-free oral polio booster vaccine made in lettuce chloroplasts confers protection against all three poliovirus serotypes. Plant. Biotechnol. J..

[52] Van Eerde A., Gottschamel J., Bock R., Hansen K.E.A., Munang’andu H.M., Daniell H., Liu Clarke J. (2019). Production of tetravalent dengue virus envelope protein domain III based antigens in lettuce chloroplasts and immunologic analysis for future oral vaccine development. Plant. Biotechnol. J..

[53] Tregoning J.S., Nixon P., Kuroda H., Svab Z., Clare S., Bowe F., Fairweather N., Ytterberg J., van Wijk K.J., Dougan G. (2003). Expression of tetanus toxin Fragment C in tobacco chloroplasts. Nucleic Acids Res..

[54] Lakshmi P.S., Verma D., Yang X., Lloyd B., Daniell H. (2013). Low cost tuberculosis vaccine antigens in capsules: Expression in chloroplasts, bio-encapsulation, stability and functional evaluation in vitro. PLoS ONE.

[55] Ward B.J., Makarkov A., Séguin A., Pillet S., Trépanier S., Dhaliwall J., Libman M.D., Vesikari T., Landry N. (2020). Efficacy, immunogenicity, and safety of a plant-derived, quadrivalent, virus-like particle influenza vaccine in adults (18–64 years) and older adults (≥65 years): Two multicentre, randomised phase 3 trials. Lancet.

[56] Ward B.J., Gobeil P., Séguin A., Atkins J., Boulay I., Charbonneau P.-Y., Couture M., D’Aoust M.-A., Dhaliwall J., Finkle C. (2020). Phase 1 trial of a Candidate Recombinant Virus-Like Particle Vaccine for Covid-19 Disease Produced in Plants. medRxiv.

[57] Capell T., Twyman R.M., Armario-Najera V., Ma J.K., Schillberg S., Christou P. (2020). Potential Applications of Plant Biotechnology against SARS-CoV-2. Trends Plant. Sci..

[58] Rybicki E.P. (2020). Plant molecular farming of virus-like nanoparticles as vaccines and reagents. Wiley Interdiscip. Rev. Nanomed. Nanobiotechnol..

[59] Vamvaka E., Arcalis E., Ramessar K., Evans A., O’Keefe B.R., Shattock R.J., Medina V., Stoger E., Christou P., Capell T. (2016). Rice endosperm is cost-effective for the production of recombinant griffithsin with potent activity against HIV. Plant. Biotechnol. J..

[60] Juarez P., Presa S., Espi J., Pineda B., Anton M.T., Moreno V., Buesa J., Granell A., Orzaez D. (2012). Neutralizing antibodies against rotavirus produced in transgenically labelled purple tomatoes. Plant. Biotechnol. J..

[61] Nandi S., Yalda D., Lu S., Nikolov Z., Misaki R., Fujiyama K., Huang N. (2005). Process development and economic evaluation of recombinant human lactoferrin expressed in rice grain. Transgenic Res..

[62] Takaiwa F., Wakasa Y., Hayashi S., Kawakatsu T. (2017). An overview on the strategies to exploit rice endosperm as production platform for biopharmaceuticals. Plant. Sci..

[63] Takaiwa F., Yang L., Takagi H., Maruyama N., Wakasa Y., Ozawa K., Hiroi T. (2019). Development of Rice-Seed-Based Oral Allergy Vaccines Containing Hypoallergenic Japanese Cedar Pollen Allergen Derivatives for Immunotherapy. J. Agric. Food Chem..

[64] Hofbauer A., Melnik S., Tschofen M., Arcalis E., Phan H.T., Gresch U., Lampel J., Conrad U., Stoger E. (2016). The Encapsulation of Hemagglutinin in Protein Bodies Achieves a Stronger Immune Response in Mice than the Soluble Antigen. Front. Plant. Sci..

[65] Van Zyl A.R., Meyers A.E., Rybicki E.P. (2017). Development of plant-produced protein body vaccine candidates for bluetongue virus. BMC Biotechnol..

[66] Cai J., Wen R., Li W., Wang X., Tian H., Yi S., Zhang L., Li X., Jiang C., Li H. (2018). Oil body bound oleosin-rhFGF9 fusion protein expressed in safflower (Carthamus tinctorius L.) stimulates hair growth and wound healing in mice. BMC Biotechnol..

[67] Qiang W., Zhou T., Lan X., Zhang X., Guo Y., Noman M., Du L., Zheng J., Li W., Li H. (2018). A new nanoscale transdermal drug delivery system: Oil body-linked oleosin-hEGF improves skin regeneration to accelerate wound healing. J. Nanobiotechnol..

[68] Chikwamba R.K., Scott M.P., Mejía L.B., Mason H.S., Wang K. (2003). Localization of a bacterial protein in starch granules of transgenic maize kernels. Proc. Natl. Acad. Sci. USA.

[69] Dauvillee D., Delhaye S., Gruyer S., Slomianny C., Moretz S.E., d’Hulst C., Long C.A., Ball S.G., Tomavo S. (2010). Engineering the chloroplast targeted malarial vaccine antigens in Chlamydomonas starch granules. PLoS ONE.

[70] Vitale A., Raikhel N. (1999). What do proteins need to reach different vacuoles?. Trends Plant. Sci..

[71] Peters J., Stoger E. (2011). Transgenic crops for the production of recombinant vaccines and anti-microbial antibodies. Hum. Vaccines.

[72] Arcalis E., Ibl V., Peters J., Melnik S., Stoger E. (2014). The dynamic behavior of storage organelles in developing cereal seeds and its impact on the production of recombinant proteins. Front. Plant. Sci..

[73] Canonica G.W., Passalacqua G. (2003). Noninjection routes for immunotherapy. J. Allergy. Clin. Immunol..

[74] Takagi H., Hiroi T., Hirose S., Yang L., Takaiwa F. (2010). Rice seed ER-derived protein body as an efficient delivery vehicle for oral tolerogenic peptides. Peptides.

[75] Herman E.M., Larkins B.A. (1999). Protein storage bodies and vacuoles. Plant. Cell.

[76] Argos P., Pedersen K., Marks M.D., Larkins B.A. (1982). A structural model for maize zein proteins. J. Biol. Chem..

[77] Llop-Tous I., Madurga S., Giralt E., Marzabal P., Torrent M., Ludevid M.D. (2010). Relevant elements of a maize gamma-zein domain involved in protein body biogenesis. J. Biol. Chem..

[78] Shewry P.R., Napier J.A., Tatham A.S. (1995). Seed Storage Proteins: Structures and Biosynthesis. Plant. Cell.

[79] Woo Y.M., Hu D.W., Larkins B.A., Jung R. (2001). Genomics analysis of genes expressed in maize endosperm identifies novel seed proteins and clarifies patterns of zein gene expression. Plant. Cell.

[80] Geli M.I., Torrent M., Ludevid D. (1994). Two Structural Domains Mediate Two Sequential Events in [gamma]-Zein Targeting: Protein Endoplasmic Reticulum Retention and Protein Body Formation. Plant. Cell.

[81] Torrent M., Llompart B., Lasserre-Ramassamy S., Llop-Tous I., Bastida M., Marzabal P., Westerholm-Parvinen A., Saloheimo M., Heifetz P.B., Ludevid M.D. (2009). Eukaryotic protein production in designed storage organelles. BMC Biol..

[82] Heifetz P.B., Royo B.L., Luna P.M., Virgili M.B., Mugica M.D.L., Quetglas M.T., O’Connor K.J., Bergwerf R.P., Tous M.I.I. (2015). Production of Biologically Active Proteins.

[83] Ludevid D., Torrent M., Lasserre-Ramassamy S. (2004). Production of Peptides and Proteins by Accumulation in Plant Endoplasmic Reticulum-Derived Protein Bodies.

[84] Torrent M., Llop-Tous I., Ludevid M.D. (2009). Protein body induction: A new tool to produce and recover recombinant proteins in plants. Methods Mol. Biol..

[85] Guo X., Yuan L., Chen H., Sato S.J., Clemente T.E., Holding D.R. (2013). Nonredundant function of zeins and their correct stoichiometric ratio drive protein body formation in maize endosperm. Plant. Physiol..

[86] Whitehead M., Ohlschlager P., Almajhdi F.N., Alloza L., Marzabal P., Meyers A.E., Hitzeroth I.I., Rybicki E.P. (2014). Human papillomavirus (HPV) type 16 E7 protein bodies cause tumour regression in mice. BMC Cancer.

[87] Fernandez-Carneado J., Kogan M.J., Castel S., Giralt E. (2004). Potential peptide carriers: Amphipathic proline-rich peptides derived from the N-terminal domain of gamma-zein. Angew. Chem. Int. Ed. Engl..

[88] Kogan M.J., Dalcol I., Gorostiza P., Lopez-Iglesias C., Pons M., Sanz F., Ludevid D., Giralt E. (2001). Self-assembly of the amphipathic helix (VHLPPP)8. A mechanism for zein protein body formation. J. Mol. Biol..

[89] Schwestka J., Tschofen M., Vogt S., Marcel S., Grillari J., Raith M., Swoboda I., Stoger E. (2020). Plant-derived protein bodies as delivery vehicles for recombinant proteins into mammalian cells. Biotechnol. Bioeng..

[90] De Virgilio M., De Marchis F., Bellucci M., Mainieri D., Rossi M., Benvenuto E., Arcioni S., Vitale A. (2008). The human immunodeficiency virus antigen Nef forms protein bodies in leaves of transgenic tobacco when fused to zeolin. J. Exp. Bot..

[91] Frandsen G.I., Mundy J., Tzen J.T. (2001). Oil bodies and their associated proteins, oleosin and caleosin. Physiol. Plant..

[92] Ling H. (2007). Oleosin fusion expression systems for the production of recombinant proteins. Biologia.

[93] Wahlroos T., Soukka J., Denesyuk A., Wahlroos R., Korpela T., Kilby N.J. (2003). Oleosin expression and trafficking during oil body biogenesis in tobacco leaf cells. Genesis.

[94] Tzen J.T.C. (2012). Integral Proteins in Plant Oil Bodies. Int. Sch. Res. Not. Bot..

[95] Parmenter D.L., Boothe J.G., van Rooijen G.J.H., Yeung E.C., Moloney M.M. (1995). Production of biologically active hirudin in plant seeds using oleosin partitioning. Plant. Mol. Biol..

[96] Van Rooijen G.J.H., Motoney M.M. (1995). Plant Seed Oil-bodies as Carriers for Foreign Proteins. Nat. Biotechnol..

[97] Liu J., Selinger L.B., Cheng K., Beauchemin K.A., Moloney M.M. (1997). Plant seed oil-bodies as an immobilization matrix for a recombinant xylanase from the rumen fungus Neocallimastix patriciarum. Mol. Breed..

[98] Nykiforuk C.L., Boothe J.G., Murray E.W., Keon R.G., Goren H.J., Markley N.A., Moloney M.M. (2006). Transgenic expression and recovery of biologically active recombinant human insulin from Arabidopsis thaliana seeds. Plant. Biotechnol. J..

[99] Qiang W., Gao T., Lan X., Guo J., Noman M., Li Y., Guo Y., Kong J., Li H., Du L. (2020). Molecular Pharming of the Recombinant Protein hEGF-hEGF Concatenated with Oleosin Using Transgenic Arabidopsis. Genes.

[100] Yi S., Yang J., Huang J., Guan L., Du L., Guo Y., Zhai F., Wang Y., Lu Z., Wang L. (2015). Expression of bioactive recombinant human fibroblast growth factor 9 in oil bodies of Arabidopsis thaliana. Protein Expr. Purif..

[101] Bundo M., Shi X., Vernet M., Marcos J.F., Lopez-Garcia B., Coca M. (2019). Rice Seeds as Biofactories of Rationally Designed and Cell-Penetrating Antifungal PAF Peptides. Front. Plant. Sci..

[102] Montesinos L., Bundo M., Izquierdo E., Campo S., Badosa E., Rossignol M., Montesinos E., San Segundo B., Coca M. (2016). Production of Biologically Active Cecropin A Peptide in Rice Seed Oil Bodies. PLoS ONE.

[103] Chatrenet B., Chang J.Y. (1993). The disulfide folding pathway of hirudin elucidated by stop/go folding experiments. J. Biol. Chem..

[104] Gallier S., Singh H. (2012). Behavior of almond oil bodies during in vitro gastric and intestinal digestion. Food Funct..

[105] Ahmed S., Zhou X., Pang Y., Jin L., Bao J. (2018). Improving Starch-Related Traits in Potato Crops: Achievements and Future Challenges. Starch-Stärke.

[106] Bull S.E., Seung D., Chanez C., Mehta D., Kuon J.-E., Truernit E., Hochmuth A., Zurkirchen I., Zeeman S.C., Gruissem W. (2018). Accelerated ex situ breeding of GBSS- and PTST1-edited cassava for modified starch. Sci. Adv..

[107] Mu-Forster C., Wasserman B.P. (1998). Surface Localization of Zein Storage Proteins in Starch Granules from Maize Endosperm: Proteolytic Removal by Thermolysin and in Vitro Cross-Linking of Granule-Associated Polypeptides. Plant Physiol..

[108] Ivanovsky D. (1882). Concerning the mosaic disease of the tobacco plant. St. Petersburg Acad. Imp. Sci. Bull..

[109] Kausche G.A., Ruska H. (1939). Die Struktur der “kristallinen Aggregate” des Tabakmosaikvirus-proteins. Biochem. Z.

[110] Wen A.M., Steinmetz N.F. (2016). Design of virus-based nanomaterials for medicine, biotechnology, and energy. Chem. Soc. Rev..

[111] Qian C., Liu X., Xu Q., Wang Z., Chen J., Li T., Zheng Q., Yu H., Gu Y., Li S. (2020). Recent Progress on the Versatility of Virus-Like Particles. Vaccines.

[112] Lizotte P.H., Wen A.M., Sheen M.R., Fields J., Rojanasopondist P., Steinmetz N.F., Fiering S. (2016). In situ vaccination with cowpea mosaic virus nanoparticles suppresses metastatic cancer. Nat. Nanotechnol..

[113] Murray K., Bruce S.A., Hinnen A., Wingfield P., van Erd P.M., de Reus A., Schellekens H. (1984). Hepatitis B virus antigens made in microbial cells immunise against viral infection. EMBO J..

[114] Valenzuela P., Medina A., Rutter W.J., Ammerer G., Hall B.D. (1982). Synthesis and assembly of hepatitis B virus surface antigen particles in yeast. Nature.

[115] Huzair F., Sturdy S. (2017). Biotechnology and the transformation of vaccine innovation: The case of the hepatitis B vaccines 1968-2000. Stud. Hist. Philos. Biol. Biomed. Sci..

[116] Schiller J.T., Castellsague X., Villa L.L., Hildesheim A. (2008). An update of prophylactic human papillomavirus L1 virus-like particle vaccine clinical trial results. Vaccine.

[117] Naupu P.N., van Zyl A.R., Rybicki E.P., Hitzeroth I.I. (2020). Immunogenicity of plant-produced human papillomavirus (HPV) virus-like particles (VLPs). Vaccines.

[118] Ma J.K., Drake P.M., Christou P. (2003). The production of recombinant pharmaceutical proteins in plants. Nat. Rev. Genet..

[119] Mason H.S., Lam D.M., Arntzen C.J. (1992). Expression of hepatitis B surface antigen in transgenic plants. Proc. Natl. Acad. Sci. USA.

[120] Marsian J., Lomonossoff G.P. (2016). Molecular pharming—VLPs made in plants. Curr. Opin. Biotechnol..

[121] Scotti N., Rybicki E.P. (2013). Virus-like particles produced in plants as potential vaccines. Expert Rev. Vaccines.

[122] Thuenemann E.C., Lenzi P., Love A.J., Taliansky M., Bécares M., Zuñiga S., Enjuanes L., Zahmanova G.G., Minkov I.N., Matić S. (2013). The use of transient expression systems for the rapid production of virus-like particles in plants. Curr. Pharm. Des..

[123] D’Aoust M.A., Lavoie P.O., Couture M.M., Trepanier S., Guay J.M., Dargis M., Mongrand S., Landry N., Ward B.J., Vezina L.P. (2008). Influenza virus-like particles produced by transient expression in Nicotiana benthamiana induce a protective immune response against a lethal viral challenge in mice. Plant. Biotechnol. J..

[124] Pang E.L., Peyret H., Ramirez A., Loh H.S., Lai K.S., Fang C.M., Rosenberg W.M., Lomonossoff G.P. (2019). Epitope Presentation of Dengue Viral Envelope Glycoprotein Domain III on Hepatitis B Core Protein Virus-Like Particles Produced in Nicotiana benthamiana. Front. Plant. Sci..

[125] Ponndorf D., Meshcheriakova Y., Thuenemann E.C., Dobon Alonso A., Overman R., Holton N., Dowall S., Kennedy E., Stocks M., Lomonossoff G.P. (2020). Plant-made dengue virus-like particles produced by co-expression of structural and non-structural proteins induce a humoral immune response in mice. Plant. Biotechnol. J..

[126] Stump C.T., Ho G., Mao C., Veliz F.A., Beiss V., Fields J., Steinmetz N.F., Fiering S. (2021). Remission-Stage Ovarian Cancer Cell Vaccine with Cowpea Mosaic Virus Adjuvant Prevents Tumor Growth. Cancers.

[127] Duval K.E.A., Wagner R.J., Beiss V., Fiering S.N., Steinmetz N.F., Hoopes P.J. (2020). Cowpea Mosaic Virus Nanoparticle Enhancement of Hypofractionated Radiation in a B16 Murine Melanoma Model. Front. Oncol..

[128] Dennis S.J., Meyers A.E., Guthrie A.J., Hitzeroth I.I., Rybicki E.P. (2018). Immunogenicity of plant-produced African horse sickness virus-like particles: Implications for a novel vaccine. Plant Biotechnol. J..

[129] Dennis S.J., O’Kennedy M.M., Rutkowska D., Tsekoa T., Lourens C.W., Hitzeroth I.I., Meyers A.E., Rybicki E.P. (2018). Safety and immunogenicity of plant-produced African horse sickness virus-like particles in horses. Vet. Res..

[130] Gunter C.J., Regnard G.L., Rybicki E.P., Hitzeroth I.I. (2019). Immunogenicity of plant-produced porcine circovirus-like particles in mice. Plant Biotechnol. J..

[131] Marsian J., Hurdiss D.L., Ranson N.A., Ritala A., Paley R., Cano I., Lomonossoff G.P. (2019). Plant-Made Nervous Necrosis Virus-Like Particles Protect Fish Against Disease. Front. Plant. Sci..

[132] Diamos A.G., Pardhe M.D., Sun H., Hunter J.G.L., Mor T., Meador L., Kilbourne J., Chen Q., Mason H.S. (2020). Codelivery of improved immune complex and virus-like particle vaccines containing Zika virus envelope domain III synergistically enhances immunogenicity. Vaccine.

[133] He J., Lai H., Esqueda A., Chen Q. (2021). Plant-Produced Antigen Displaying Virus-Like Particles Evokes Potent Antibody Responses against West Nile Virus in Mice. Vaccines.

[134] Pumpens P., Borisova G.P., Crowther R.A., Grens E. (1995). Hepatitis B virus core particles as epitope carriers. Intervirology.

[135] Peyret H., Gehin A., Thuenemann E.C., Blond D., El Turabi A., Beales L., Clarke D., Gilbert R.J., Fry E.E., Stuart D.I. (2015). Tandem fusion of hepatitis B core antigen allows assembly of virus-like particles in bacteria and plants with enhanced capacity to accommodate foreign proteins. PLoS ONE.

[136] Peyret H., Ponndorf D., Meshcheriakova Y., Richardson J., Lomonossoff G.P. (2020). Covalent protein display on Hepatitis B core-like particles in plants through the in vivo use of the SpyTag/SpyCatcher system. Sci. Rep..

[137] Ward B.J., Landry N., Trepanier S., Mercier G., Dargis M., Couture M., D’Aoust M.A., Vezina L.P. (2014). Human antibody response to N-glycans present on plant-made influenza virus-like particle (VLP) vaccines. Vaccine.

[138] Chung Y.H., Cai H., Steinmetz N.F. (2020). Viral nanoparticles for drug delivery, imaging, immunotherapy, and theranostic applications. Adv. Drug Deliv. Rev..

[139] Sainsbury F., Canizares M.C., Lomonossoff G.P. (2010). Cowpea mosaic virus: The plant virus-based biotechnology workhorse. Annu. Rev. Phytopathol..

[140] Shin M.D., Shukla S., Chung Y.H., Beiss V., Chan S.K., Ortega-Rivera O.A., Wirth D.M., Chen A., Sack M., Pokorski J.K. (2020). COVID-19 vaccine development and a potential nanomaterial path forward. Nat. Nanotechnol..

[141] Shukla S., Hu H., Cai H., Chan S.K., Boone C.E., Beiss V., Chariou P.L., Steinmetz N.F. (2020). Plant viruses and bacteriophage-based reagents for diagnosis and therapy. Annu. Rev. Virol..

[142] Steinmetz N.F., Lim S., Sainsbury F. (2020). Protein cages and virus-like particles: From fundamental insight to biomimetic therapeutics. Biomater. Sci..

[143] Steinmetz N.F., Cho C.F., Ablack A., Lewis J.D., Manchester M. (2011). Cowpea mosaic virus nanoparticles target surface vimentin on cancer cells. Nanomedicine.

[144] Koudelka K.J., Destito G., Plummer E.M., Trauger S.A., Siuzdak G., Manchester M. (2009). Endothelial targeting of cowpea mosaic virus (CPMV) via surface vimentin. PLoS Pathog..

[145] Berardi A., Evans D.J., Baldelli Bombelli F., Lomonossoff G.P. (2018). Stability of plant virus-based nanocarriers in gastrointestinal fluids. Nanoscale.

